# Supplementary material for: Public Health Implications of Wasting and Stunting Relationship in Children under Five Years Highly Vulnerable to Undernutrition in Guatemala: The REDAC Study
Source: Nutrients. 2022 Sep 23;14(19):3945. doi: 10.3390/nu14193945 (PMC9572142; doi:10.3390/nu14193945)
Supplement: Supplementary file 1 [file nutrients-14-03945-s001.zip › nutrients-1931422-supplementary.pdf]

Supplemental Material

Table S1. Organizations and municipalities participating in the nutritional monitoring of the REDAC study

DEPARTMENTS	ORGANIZATIONS	MUNICIPALITIES
HUEHUETENANGO 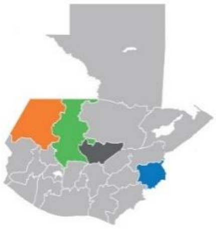	COOPI COOPERAZIONE INTERNAZIONALE<br>GVC	Colotenango<br>Malacatancito<br>San Gaspar Ixchil<br>San Ildefonso Ixtahuacán<br>San Miguel Acatán<br>San Rafael Petzal
QUICHÉ 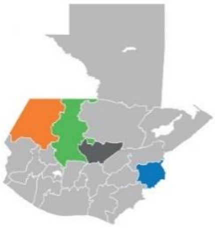	Trócaire Working for a just world.<br>Caritas Pastoral Social Diócesis de Quiché	Sacapulas<br>San Andrés Sajcabajá<br>San Pedro Jocopilas
CHIQUIMULA 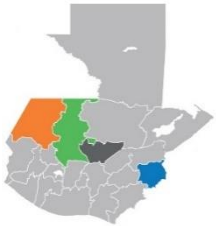	OXFAM<br>ADEL ASEDECHI	Jocotán<br>Olopa
BAJA VERAPAZ 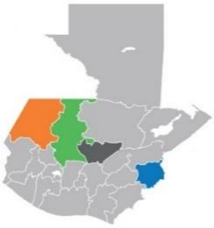	OXFAM<br>ASOCIACIÓN DE PADRES DE FAMILIA CORAZÓN DEL MAÍZ SAN MIGUEL CHICAJ BAJA VERAPAZ	Cubulco<br>Rabinal<br>San Miguel Chicaj
ALL DEPARTMENTS 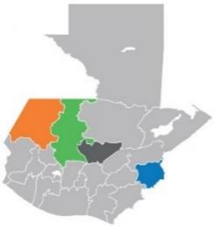	ACCIÓN CONTRA EL HAMBRE	Coordination and quality control of data collection

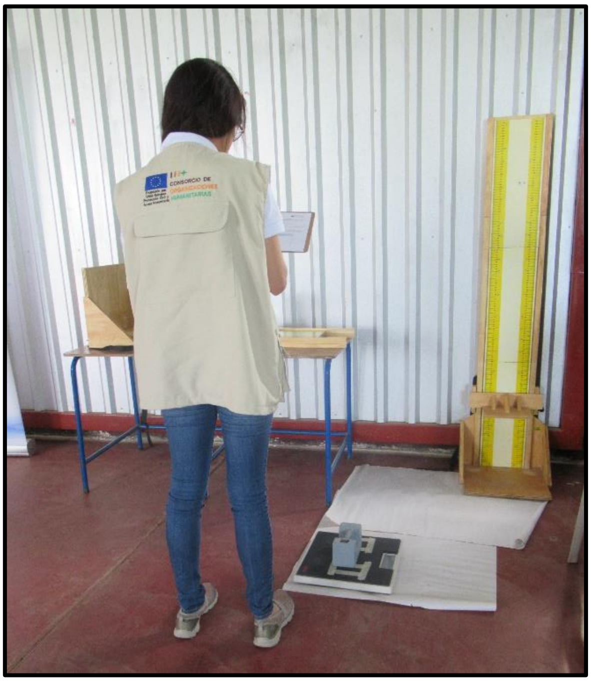

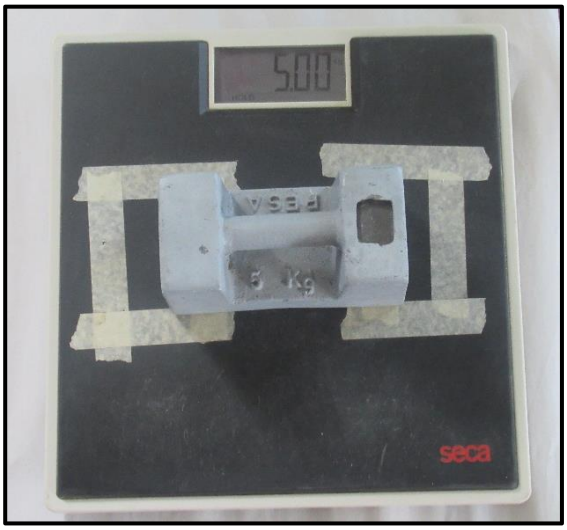

S1a. Calibration process of the scale using an object of known weight.

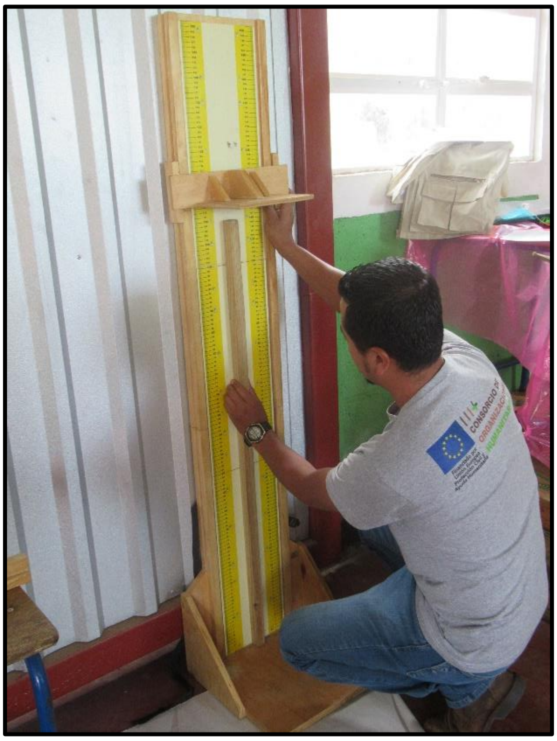

S1b. Calibration process of the measuring tallimeter using a wooden rod of known length.

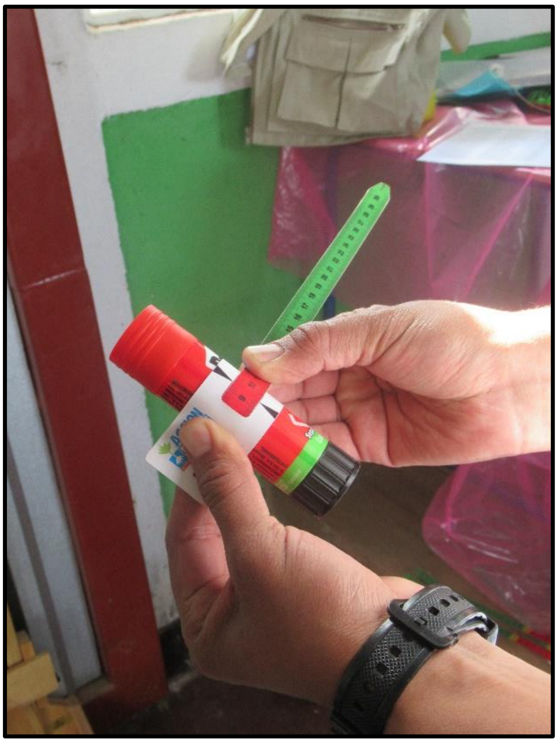

S1c. Mid-upper arm circumference (MUAC) bracelet calibration process using a rigid cylindrical object of known circumference

Figure S1. Calibration process of the anthropometric material before each data collection session

Table S2. Distribution of the sample according to age, geographic origin and sex.

	BOYS		GIRLS		TOTAL	
	N	%	N	%	N	%
< 24 months	2857	43.4%	2664	41.3%	5521	42.4%
≥ 24 months	3726	56.6%	3783	58.7%	7509	57.6%
0 – 6 months	658	10.0%	521	8.1%	1179	9.1%
6 – 12 months	781	11.9%	689	10.8%	1476	11.4%
12 – 18 months	734	11.2%	757	11.8%	1491	11.5%
18 – 24 months	684	10.4%	691	10.8%	1375	10.6%
24 – 30 months	790	12.1%	698	10.9%	1488	11.5%
30 – 36 months	640	9.8%	627	9.8%	1267	9.8%
36 – 42 months	685	10.5%	673	10.5%	1358	10.5%
42 – 48 months	616	9.4%	610	9.5%	1226	9.5%
48 – 54 months	532	8.1%	610	9.5%	1142	8.8%
54 – 60 months	435	6.6%	531	8.3%	966	7.4%

	BOYS		GIRLS		TOTAL	
**REGION**	N	%	N	%	N	%
Northeast	2781	42.2%	2777	43.1%	5558	42.7%
West	3803	57.8%	3670	56.9%	7473	57.3%
**DEPARTMENTS**	N	%	N	%	N	%
Huehuetenango	2411	36.6%	2501	38.8%	4912	37.7%
Quiché	1392	21.1%	1169	18.1%	2561	19.7%
Chiquimula	1733	26.3%	1653	25.6%	3386	26.0%
Baja Verapaz	1048	15.9%	1124	17.4%	2172	16.7%
**MUNICIPALITIES**	N	%	N	%	N	%
**Huehuetenango**						
Colotenango	398	6.0%	416	6.5%	814	6.2%
Malacatancito	395	22.8%	400	6.2%	795	6.1%
San Gaspar Ixchil	238	3.6%	235	3.6%	473	3.6%
San Ildefonso Ixtahuacán	6290	9.6%	660	10.2%	1289	9.9%
San Miguel Acatán	687	10.4%	742	11.5%	1429	11.0%
San Rafael Petzal	64	1.0%	48	0.7%	112	0.9%
**Quiché**						
Sacapulas	324	4.9%	270	4.2%	594	4.6%
San Andrés Sajcabajá	851	12.9%	738	11.4%	1589	12.2%
San Pedro Jocopilas	217	3.3%	161	2.5%	378	2.9%
**Chiquimula**						
Jocotán	1499	22.8%	1364	21.2%	2826	22.0%
Olopa	234	3.6%	289	4.5%	523	4.0%
**Baja Verapaz**						
Cubulco	418	6.3%	493	7.6%	911	7.0%
Rabinal	197	3.0%	186	2.9%	383	2.9%
San Miguel Chicaj	433	6.6%	445	6.9%	878	6.7%

Table S3. Data included in the analysis after cleaning of potential errors.

**Fixed exclusions (biologically implausible)**			
	Plausible range (z-score)	Number out-of-range data	Proportion of total recorded
Height-for-age	Between -6 y +6	511	3.9%
Weight-for-height	Between -6 y +5	14	0.11%
Weight-for-age	Between -5 y +5	98	0.75%
MUAC-for-age	Between -5 y +5	3	0.03%
**Flexible exclusions (based on the sampling distribution itself)**			
	Accepted range (z-score)	Number out-of-range data	Proportion of total recorded
Height-for-age	Between -4 y +4	105	0.81%
Weight-for-height	Between -4 y +4	27	0.21%
Weight-for-age	Between -4 y +4	76	0.58%
MUAC-for-age	Between -4 y +4	8	0.07%
**Results of the cleaning process**			
	Number of excluded data	Proportion of total recorded	Number of cases included in the analysis
Height-for-age	511	3.9%	12496
Weight-for-height	27	0.21%	12886
Weight-for-age	123	0.95%	12885
MUAC-for-age	8	0.07%	11833

MUAC: mid-upper arm circumference

Table S4. Contingency table for the classification of acute malnutrition (AM) according to two different anthropometric indicators.

		Weight-for-height (WHZ)		
		**With AM**	**Without AM**	**Total**
**Mid-upper arm circumference (MUAC)**	**With AM**	Cases: 141<br>28.3%<br>40.9%	Cases: 358<br>71.7%<br>3.1%	Cases: 499<br>100.0%<br>4.2%
	**Without AM**	Cases: 204<br>1.8%<br>59.1%	Cases: 11080<br>98.2%<br>96.9%	Cases: 11284<br>100.0%<br>95.8%
	**Total**	Cases: 345<br>2.9%<br>100.0%	Cases: 11438<br>97.1%<br>100.0%	Cases: 11783<br>100.0%<br>100.0%
**Concordance**		Kappa: 0.310; p<0.001		
**Diagnostic Efficacy***		Sensitivity: 0.409; Specificity: 0.969;<br>Accuracy: 0.952; Likelihood ratio: 0.610		

* Diagnostic efficacy of MUAC with respect to the reference classification provided by the WHZ.
